# Effect of an Active Break Intervention on Attention, Concentration, Academic Performance, and Self-Concept in Compulsory Secondary Education

**DOI:** 10.3390/ejihpe14030030

**Published:** 2024-02-22

**Authors:** Julen Maiztegi-Kortabarria, Silvia Arribas-Galarraga, Izaskun Luis-de Cos, Sebastián Espoz-Lazo, Pedro Valdivia-Moral

**Affiliations:** 1 Education and Sport Faculty (Education), University of the Basque Country (UPV/EHU), 01006 Vitoria-Gasteiz, Spain; 2 Faculty of Education, Philosophy and Anthropology, University of the Basque Country (UPV/EHU), 20018 Donostia-San Sebastián, Spain; silvia.arribas@ehu.eus (S.A.-G.); izaskun.luis@ehu.eus (I.L.-d.C.); 3 Faculty of Medical Sciences, Sciences of Physical Actvity, Sports and Health School, University of Santiago de Chile (USACH), Santiago 9170022, Chile; sebastian.espoz@usach.cl; 4 Faculty of Science Education, University of Granada (UGR), 18071 Granada, Spain; pvaldivia@ugr.es

**Keywords:** intense physical activity, academic performance, attention, concentration, academic self-concept, basic psychological needs

## Abstract

(1) Background: Society’s shift to a tech-focused era and has created a hyper-connected, sedentary lifestyle. The purpose of this study is to address two objectives: firstly, to describe and analyze the effects of an active breaks program associated with the learning of curricular content (CF-AB) on levels of attention, concentration, and academic performance (AP); secondly, to examine the relationship between intense physical activity (PA), attention, concentration, academic self-concept, basic psychological needs, and academic performance in schoolchildren who practice CF-ABs. (2) Method: A randomized controlled trial quasi-experimental pre-test/post-test study with a non-probabilistic sample included 313 secondary school students divided into intervention and control groups. The intervention, a curricular-focused academic break (CF-AB) (8 weeks, 5–10 min/session), is taken in the middle of the class and linked with the subject content. Measuring instruments: Attention Test D2, ad hoc test for the AP, self-concept AF5, Basic Psychological Needs Satisfaction Scale in General (BNSG-S), and the Global PA Questionnaire (GPAC.V2). (3) Results: Attention and concentration improved in both groups, with no significant differences. There were no significant differences in academic self-concept, but the intervention group showed higher scores in basic psychological needs. AP correlated positively with concentration, academic self-concept, and physical activity. A proportion of 20% of the variance of AP in spelling is explained by the regression model. Students who improved the most in AP practiced intense PA outside school, with good self-concept and satisfactory social relationships. Although concentration was related to AP, it did not explain the improvement. (4) Conclusion: CF-ABs may have a positive impact on attention and AP, with socioemotional factors and PA playing an important role in this effect. (5) Limitations and Future Research: The relationship between PA performed in class and AP should be considered with caution due to the multifactorial nature of AP. Future research should consider the number of sessions per week, the prolongation of the same during the school year, the intensity and duration of the activity, and the intervention type of active breaks. In addition, attention should be paid to possible incident factors in AP related to personal and social variables.

## 1. Introduction

Since the 1970s, the exponential development of technology has been introduced into the classroom, evolving toward the so-called technological paradigm in the 21st century. The direct consequences of this change are projected on the methodologies used in the teaching and learning processes in schools, and increasingly, a hyperconnected-to-screens society with a sedentary lifestyle is created [1].

Several studies show that older adolescents show low levels of physical activity (PA) due to a greater interest and dedication to sedentary leisure activities such as the use of screens [2], a phenomenon referring to the free time spent watching television, using computers, and playing video games, which some authors have called a “technological sedentary lifestyle” [3].

Alerted by the change in habits and its consequences, the World Health Organization [4], among other recommendations, points to the importance of moderate and intense PA as elements that “boost health”.

Among the drawbacks of sedentary habits, some affect the cognitive processes necessary for proper academic performance (AP). According to Ortiz [5], cognition refers to the act or process of knowing and the psychological processes involved in perception, attention, memory, recall, and thinking, which play a fundamental role in the cognitive and higher capacities of human beings, i.e., in rational processes (thought, language, intelligence, and creativity). Through cognitive processes or cognitive configuration, neural networks and circuits are created, enabling the acquisition of new knowledge. According to this author, cognitive processes are configured on the basis of a double structure: on the one side, simple cognitive processes that are divided into sensory processes (sensations, perception, attention, and concentration) and representational processes (memory, imagination and dreaming); on the other side, the complex or higher cognitive processes classified as rational processes (intelligence, language, and thought). These complex cognitive processes are more sophisticated than the simple processes and are formed from the simple processes.

In this regard, there is evidence suggesting that PA can improve cognitive functions and promote better well-being in individuals, as well as benefit the academic performance (AP) of school children [6,7]. Along the same line, Fenesi et al. [8] indicate the benefits of PA practice on the neurocognitive and psychoemotional development of students. The authors report that PA has direct and immediate effects on the brain (increased blood flow, increased neurotransmitters that improve cognitive functioning). Similarly, they suggest that the introduction of PA in the classroom increases feelings of joy and motivation to learn, along with improved classroom behavior.

According to Tarabini [9], schools and/or academic centers are considered specialized institutions for transmitting knowledge, skills, and abilities, together with shaping attitudes, dispositions, and characteristics that favor the comprehensive development of personal identity. Therefore, it is interesting to highlight the importance of stimulating and enriching environments that favor stimulation of simple or sensory cognitive processes (sensations, perception, attention, and concentration) as they are the basis of a correct cognitive process [5] that facilitate and favor students’ AP.

According to Steinmayr et al. [10], AP is a widely accepted measure that assesses the extent to which students reach the educational objectives set out in curricula. It seeks to determine the level of knowledge and skills that students have acquired in different academic areas, in this case, in the school context. It is important to consider that, as Nieto Martín [11] indicates, personal variables (cognitive variables, study habits, motivation, attention, concentration, self-concept, emotions) and social variables (social environment, socio-economic context, and socio-democratic dimensions) make up AP’s multifactorial nature.

Among the authors that took the association between PA practice and AP into account, Andrades-Suarez et al. [12] analyzed the effect of PA on AP and executive functions of the brain, concluding that PA has a positive influence on AP and executive function variables. This influence mainly translates into improved performance in mathematics and/or reading and memory and attention. The results of the study suggest that regular practice of PA can lead to significant academic and cognitive benefits, which highlights the importance of promoting PA as an integral part of students’ development and learning. Similarly, Giner et al. [13] observed a correlation between PA practice and AP in primary and secondary school students (mean age = 12.37). Trullén [14] investigated the factors that influence AP by analyzing the impact of out-of-school PA practice on AP. He concluded that out-of-school PA has a positive impact on AP, notably in girls and with respect to the subject of mathematics. In their systematic review about PA interventions and AP, Alvarez-Bueno et al. [15] suggest that PA-based interventions significantly benefit AP as well as classroom behavior. Similarly, in their systematic review about the relationship between PA practice and AP in schoolchildren, Chacón-Cubero et al. [16] reported that PA improves AP, and with greater improvements, the greater the volume and intensity of PA performed.

There is also a large body of research that has analyzed the impact of active breaks on AP, although the results point in opposite directions. Active breaks (AB) are breaks during lessons, lasting 5 to 10 min, for moderate or vigorous physical activity [17,18]. Research by Bartholomew et al. [19] (mathematics, language arts, and science), Fiorilli et al. [20] (mathematics), Macdonald et al. [21] (mathematics and reading), Magistro et al. [22] (arithmetic reasoning), Mavilidi et al. [23], and Mok et al. [24] reported significant differences between groups following AB interventions. In contrast, research by Arribas-Galarraga et al. [17] (spelling), Donnelly et al. [25] (mathematics, reading comprehension, spelling), Egger et al. [26] (spelling and reading), Erwin et al. [27] (mathematics and reading), Fedewa et al. [28] (mathematics), Mullender-Wijnsma et al. [29] (reading, spelling, mathematics, mathematics speed), Raney et al. [30], Snyder et al. [31], Solberg et al. [32], van den Berg et al. [33], and Young-Jones et al. [34] showed no significant differences between the groups. In addition, Mullender-Wijnsma et al. [35] found a significant effect between the AB and control groups in some of the groups, but not in all groups. This controversy invites further research.

Thus, when determining the variables that affect AP, studying attention, concentration, self-concept, and their relationship with others as a source of motivation is of interest.

Some authors present the attention variable as a key element in the activation of cognitive processes such as perception and memory [36]. According to Sohlberg and Mateer’s Clinical Model of Attention [37,38], attention is constituted following a hierarchical model, with the lower levels being more complex processes that require the correct functioning of higher levels. According to this hierarchical model of attention, the highest level is arousal, or the ability to be awake and alert, and it consists of the general activation of the organism to follow orders or stimuli. Focused Attention is the ability to respond directly to specific visual, auditory, or tactile stimuli at the second level. Sustained Attention is the ability to maintain consistent behavioral responses during continuous or repetitive activities. Selective Attention is the ability to maintain a behavior or cognitive set when faced with distractions or competing stimuli. Alternate Attention is the ability to shift the focus of attention and have mental flexibility to switch between tasks with different cognitive requirements. Finally, at the lowest level and therefore the most complex, is divided attention, or an individual’s ability to respond simultaneously to multiple tasks.

Regarding AB incidence on the attention variable, research by Ma et al. [39] and Magistro et al. [22] indicated significant differences between groups after the intervention in favor of the experimental group. Similarly, Contreras Jordán et al. [40] analyzed the incidence in the intervention group, pointing to a significant increase in attention levels after ABs. Research by Arribas-Galarraga et al. [17] observed significant differences after the intervention based on curricular-focused breaks (CF-ABs) in the intervention group, but not in comparison with the control group. In the study of Bartholomew et al. [19], they analyzed the incidence of CF-ABs on attention after the implementation of the program Texas I-CAN! in 3rd and 4th grade elementary school students. The results indicate that attention increased after active classes, but decreased after sedentary classes. Janssen et al. [41] observed that moderate and vigorous intensity PA breaks interventions significantly improved attention scores (*p* < 0.001) compared to the control condition. In their systematic review, Pastor-Vicedo et al. [42] analyzed the characteristics of activity intensity, duration, and the type of intervention carried out through active breaks to identify which have a greater impact on attention, concentration, and AP. The results indicated that active breaks are a valid strategy to obtain a higher cognitive performance, which has an impact on higher levels of attention, concentration, AP, and motivation. In addition, the studies reviewed in the systematic review point to differences in results due to the variety of interventions (duration, type, and intensity). The results indicate that the duration of the interventions is more effective when they are pauses ranging from 5 to 10 min. As for the intensity of PA, the results indicate that activities performed at vigorous intensity report greater benefits.

Concentration is a variable closely linked to attention. Ortiz [5] defines concentration as the ability to block irrelevant information, focus on what is important, and maintain this ability for long periods of time. Students encounter multiple simultaneous stimuli in the classroom, external and internal, which they are often unable to process. Acts involving surprise, novelty, or the satisfaction of a need will capture their attention. Thus, teachers’ intervention as facilitators of classroom dynamics that encourage active student participation can be considered relevant.

When reviewing research on the relationship between concentration and AB, no consensus has been found. The study by Contreras Jordán et al. [40], which analyzed the effect of AB in the intervention group without a control group, showed significant differences in the concentration variable. Similarly, research by Peiris et al. [43] indicates significant differences after the intervention in the intervention group and between the control and intervention groups. However, in the Scholz et al. [44] study, the difference between groups was not significant after the first six months of intervention, but it was significant at the beginning and end of the second year of intervention. In the study by Arribas-Galarraga et al. [17], the level of concentration improved significantly in the intervention group, but the results did not show significant differences between the intervention and control groups after the application of CF-ABs. Similarly, Mercader [45] observed no significant differences between the groups after a mindfulness-based intervention.

The self-concept is another variable associated with AP. Shavelson et al. [46] defined self-concept as the perception of oneself formed from experiences and relationships with the environment, and it is highly linked to environmental reinforcement and significant others. According to Rosenberg [47], a generic self-concept is made up of the set of images, thoughts, and feelings that a person has of him or herself. According to Shavelson et al.’s [46] model of self-concept, self-concept is a multidimensional and hierarchical variable. At the top of the hierarchy is the global self-concept, which is stable. At the bottom are more situation-dependent domains, which are less stable. Thus, a distinction is made between academic self-concept (languages, social studies, mathematics, science) and non-academic self-concept, which includes the sub-dimensions of social self-concept (peers and significant others), emotional self-concept (particular emotional states), and physical self-concept (physical abilities and physical appearance).

Numerous studies have analyzed the relationship between academic self-concept and academic performance. Veas et al. [48] analyzed the correlation between AP and academic self-concept in 1400 students (mean age = 12.5) and found a significant correlation between AP and academic self-concept. Carcamo et al. [49] analyzed the relationship between AP and self-concept of ability in mathematics and language in 406 fourth- and fifth- grade students. The results indicate that self-concept, achievement expectations, and age are significant factors explaining performance in both mathematics and language. These three elements had an impact on students’ performance in both subjects, suggesting that their self-perception, expectations, and age influence their academic performance in these specific areas. García Perales et al. [50] indicated that the general and academic self-concept of primary school students positively correlates with academic performance, indicating that a positive self-perception influences better school performance. In the systematic review by Mansilla Chacon et al. [51], the relationship between self-concept and AP was analyzed. In their study, it can be observed that primary and secondary school students with a high academic self-concept are linked to better AP, especially in adolescents.

On the other hand, Rojas-Jimenez et al. [52] reported a positive and direct association between self-concept and regular PA practice. This means that people with a high self-concept tend to participate more frequently in physical activities, whereas those with a lower self-concept tend to perform less PA. This suggests a bidirectional relationship, so self-concept may influence a person’s physical activity habits, and in turn, regular physical activity may have a positive impact on a person’s self-perception.

In terms of other factors that may influence AP, motivation should be considered. Specifically, Self-Determination Theory [53] is a general theory of motivation and personality that explores the extent to which people perform their actions with a high level of reflection and commitment, acting with a sense of choice. This theory comprises four mini-theories: Cognitive Appraisal Theory, Organic Integration Theory, Causal Orientations Theory, and Basic Needs Theory. According to Basic Needs Theory [54,55], the dimensions necessary for the correct performance of students’ academic functions are autonomy (perceiving oneself as the origin of one’s behavior), competence (feeling effective in interactions with social environment and feeling the opportunity to exercise one’s abilities), and relatedness (feeling connected and accepted by other people).

Recently, Wang et al. [56] indicated that satisfaction or not of basic psychological needs predicts AP. Studies such as Buzzai et al. [57] have shown positive correlations of AP with the dimensions of autonomy satisfaction, relationship satisfaction, and competence satisfaction in a sample of adolescent students (mean age = 16.19). Furthermore, the findings of Liu et al. [58] revealed that students’ class participation is positively correlated with the dimensions of competence and relatedness, and that class participation is positively correlated with AP.

Based on the literature reviewed, the evidence indicates that society is moving towards higher and higher levels of sedentary lifestyles with negative repercussions that affect the physical and psychological health of individuals, as well as the cognitive processes associated with AP. This paradigm shift equally affects the student population, who spend most of their school time in sedentary activities focused on the transmission of knowledge, skills, and abilities. Therefore, it is necessary to promote methodologies that promote the transmission of knowledge, skills, and abilities through active methodologies.

The present study proposes an intervention based on the introduction of PA in the classroom as a teaching–learning method such as CF-ABs. The scientific literature invites us to think of CF-ABs as a valid strategy to promote cognitive [6,7,8] and physical activation in a society tending to sedentary habits. By implementing CF-ABs, sedentary teaching routines are changed, allowing moderate-vigorous intensity PA to be performed while working on academic content.

In order to describe the incidence of CF-ABs on attention and concentration and the relationship between intense physical activity (PA) and AP, the present study considers the multifactorial approach to AP. This model takes into account the multifactorial nature of AP, pointing out personal variables (attention, concentration, motivation, and self-concept) and social variables (social environment of the classroom) as variables that affect students’ AP.

For this reason, the present study considers it opportune to address two objectives: (1) to describe and analyze the effects of the application of a program of active breaks associated with learning of curricular content (CF-AB) on the levels of attention, concentration and academic performance; (2) to analyze the relationship between intense physical activity (PA), attention, concentration, academic self-concept, basic psychological needs, and academic performance in schoolchildren who practice CF-ABs.

## 2. Materials and Methods

### 2.1. Participants

A randomized controlled trial quasi-experimental pre-test post-test study with a non-probabilistic incidental sample group consisted of students in Compulsory Secondary Education (ESO) from several schools in the Basque Autonomous Community (Spain), who studied Spanish Language and Literature and Basque Language. A total of 313 participants voluntarily participated in the study, aged 12–15 (mean age = 12.7, SD = 0.732), of whom 142 were girls (45.4%), 157 were boys (50.2%), and 14 were non-binary people (4.5%). The participants were divided by a random drawing process into two class groups: control group (*n* = 114) and experimental group (*n* = 199).

### 2.2. Instruments

The variables attention and concentration, self-concept, basic psychological needs, and PA habits of the students were measured using standardized tests according to their numerous uses in studies of the same line. To collect data on students’ AP, ad hoc tests were created to measure the work content in the classroom using the cards created for the CF-AB intervention. The instruments used to measure the variables studied in this research are presented below:

Attention and concentration
  - Attention Test D2. The Spanish version [59] was used. This test measures selective attention and mental concentration, individually and collectively, in a population aged 8–88 years. Test D2 consists of 14 rows made up of 47 characters, 658, represented by the letters “d” or “p”. Each letter may have one or two dashes placed in pairs or individually on its top or bottom. The test is based on reviewing the content of each line, left to right, in a set time of 20 s and pointing to any letter “d” containing two dashes, i.e., the relevant element. The resulting score areas are as follows: TR, total responses, number of elements attempted in the 14 lines; TA, total hits, number of relevant correct elements; O, omissions, number of relevant elements attempted but not marked; C, commissions, number of irrelevant elements marked; TOT, total test effectiveness, i.e., TR (O + C); CON, concentration index or TA C; TR+, line with the highest number of items attempted; TR, line with the lowest number of elements attempted; and VAR, variance index or difference (TR+) (TR). The estimated time for completion, including application instructions, is 8–10 min.

The Spanish version presents high reliability for the main dimensions (TR, TA, O, and C), with reliability coefficients ranging from 0.86 to 0.99.

Self-concept
  - Self-concept AF5 [60]. The test measures general self-concept and its subdivisions, academic dimension (items 1, 6, 11, 16, 21, 26), emotional (3, 8, 13, 18, 18, 23, 18), family (items 4, 9, 14, 19, 24, 29), social (items 2, 7, 17, 12, 27), and physical (5, 10, 15, 20, 25, 30) dimensions. The test consists of responding to 22 statements indicating a degree of agreement between 1 and 99. The reliability coefficient in relation to the subdimension is as follows: academic 0.835; emotional 0.79; family 0.782; social 0.792; and physical 0.771.
Basic Psychological Needs
  - Basic Psychological Needs Satisfaction Scale in General (BNSG-S) [61]. The test consists of 26 items measuring the subdimensions of autonomy (items 1, 7, 13), relationship subdimension (items 2, 5, 6 (−), 8, 10, 14 (−), 16) and competence dimension (items 3 (−), 4, 9, 11, 12 (−), and 15 (−)). They are answered according to the conformity of the statement on a Likert scale (1 = not true at all; up to 7 = completely true). The reliability coefficients of the sub-dimensions are 0.72 for the competence dimension, 0.81 for autonomy, and 0.82 for relatedness.
Physical Activity
  - Global Physical Activity Questionnaire (GPACv2) [62]. This questionnaire consists of 16 items to assess PA in areas such as work (school), travel, and leisure time. To measure PA, it considers intensity, frequency, and duration. Results determine whether someone is active (150+ minutes/week) or insufficiently active. The reliability coefficient 0.8 and validity is 0.3.
Academic performance
  - Academic performance (Spanish Language and Literature). An ad hoc test was used to measure knowledge of the Spanish language and, in particular, performance related to simple spelling and grammatical aspects. The academic content of the test is developed in consensus with the teaching staff specializing in the subject of Spanish, under the supervision of experts in the field. The test measures students’ academic performance on syllable identification, stressed syllables, stress (palabras llanas, agudas, and esdrujulas—words stressed on the last, penultimate and antepenultimate syllable, stress, and interrogative stress), nouns and nouns, adjectives, articles, pronouns, verb conjugation, prepositions, connectors, and spelling (use of B, V, H, G-J, and Y-LL). Scores range from 0 (minimum possible knowledge) to 287 (maximum possible knowledge).
  - Academic performance (Basque—Euskara). An ad hoc test was used to measure knowledge of the Basque language and, specifically, performance in relation to simple spelling and grammatical aspects. The academic content of the test agreed upon with the teaching staff specializing in the subject, under the supervision of experts in the field. The test measures students’ academic performance on spelling (sibilants: s, z, ts, tx, tz; use of H), ergative: nor ala nork?, adjectives (adjective, pseudonym), concordance, demonstrative, and verbs (scores range from 0 (minimum possible knowledge) to 287 (maximum possible knowledge).

### 2.3. Intervention Procedure and Program

First, a protocol of action was drawn up in accordance with regulations established by the Ethics Committee for Research involving Human Beings of the University of the Basque Country CEISH-UPV/EHU, BOPV 32, (17 February 2014). After obtaining the validation of the Ethics Committee (Ref. M10_2021_101), initial contact was made with the schools and their boards.

After acceptance, the conditions of the informed consent protocol were specified to each student and their legal representatives.

The selection of participants in the research was carried out by convenience, considering that there were two (control and intervention) in each center. As for the distribution of class groups, it was performed with a draw.

The intervention program, which lasted 8 weeks, focused on the application of curriculum-focused active breaks or curricular active breaks (CF-ABs), which sought to physically activate the pupils and, at the same time, to encourage revision or internalization of certain orthographic content specific to the subject in which it was being developed: Spanish Language and Literature, or the subject of Basque Language—Euskera.

To conduct this research, a series of simple activities were designed and organized in the form of worksheets [63,64], specifically designed to be applied in the classroom by non-expert teachers or teachers with no relation to physical activity; in other words, they were designed to be applied by any teacher. In this case, worksheets combine physical activity of moderate to vigorous intensity with aspects related to spelling and rules of use of the Spanish and Basque languages (Euskera). The duration of the active breaks is 5–10 min, and it is recommended to implement them toward the middle of the session.

Data collection of attention, concentration, and academic performance variables was carried out in two phases for both the control and intervention groups (pre-test and post-test). The data collection of the variables Basic Psychological Needs (BNSG-S), Self-Concept AF5, and Global Physical Activity Questionnaire (GPACv2) in both the control and intervention groups was collected in the pre-test phase.

**Figure 1:**
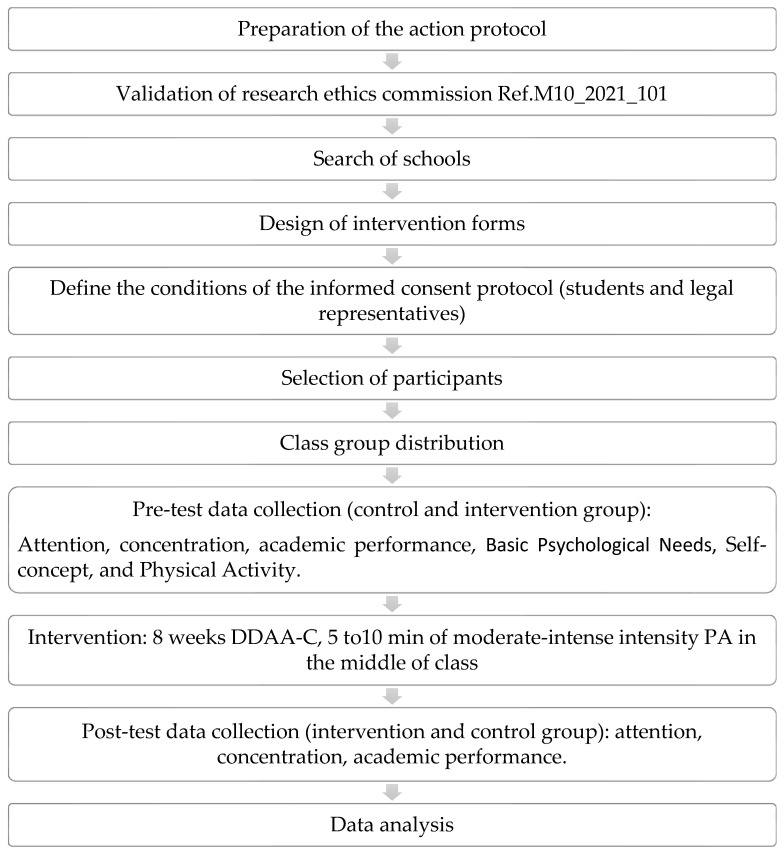
Intervention procedure and program.

Figure 1 shows the intervention procedure and the program carried out during the research.

### 2.4. Data Analysis

In line with the objective established in this study, descriptive statistical analysis was performed for the following variables: attention, concentration, academic performance, academic self-concept, the “relationship with others” dimension of basic psychological needs, and moderate or intense physical activity (measured in hours per week) of the participants. To explore the relationships between these variables, parametric analysis was employed, namely bivariate correlations and a multiple linear regression model. Statistical analysis was conducted using IBM SPSS Statistics 28 software.

## 3. Results

### 3.1. Attention and Concentration

After the intervention, the results of the D2 test, related to the variables of attention and concentration, are shown in Table 1. As it can be seen, in the range of 0–685 for attention, the results obtained after descriptive tests of this variable indicate that the experimental group obtained a greater increase (improvement of 38.08 average points) than the control group (36.18 average points). Parametric tests confirmed that the differences after the CF-AB intervention in the attention variable were significant (*p* < 0.001) in both groups. Nevertheless, the differences between groups after the intervention were not significant (Z = −0.951; *p* = 0.342).

As for the concentration variable, from 0 to 299, both groups improved their initial scores favoring the control group (improvement of 15.62 average points) compared with the experimental group (improvement of 15.2 average points). Differences after implementation of the CF-AB program indicate significant improvements (*p* < 0.001) in both groups. After completion of the intervention, the difference in concentration of the control group compared to the experimental group was not statistically significant (Z = −1.130, *p* = 0.259).

### 3.2. Academic Performance

The results of academic performance test are detailed in Table 2. After the active breaks intervention (CF-AB), both groups decreased in the AP score.

### 3.3. Academic Self-Concept, Relationship, and PA by Group

The results of the academic self-concept, relationship and PA tests are presented in Table 3. Academic self-concept scores of the control group (M = 6.804, SD = 1.673) were similar to those of the experimental group (M = 6.847, SD = 2.03), and no differences were observed between the two groups. In terms of basic psychological needs, the experimental group (M = 23.57, SD = 5.905) scored an average 6.55 points more than the control group (M = 17.02, SD = 8.640). According to the amount of moderate or intense PA performed by the students, the data indicate no difference between the control group and the intervention group.

### 3.4. Correlation: Academic Performance AP (Post), Attention (Post), Concentration (Post), Basic Psychological Needs NPB (Relationship), Self-Concept, Intense Physical Activity (PA) Outside School

Table 4 shows correlation between academic performance (AP) (post), attention (post), concentration (post), Basic Psychological Needs NPB (Relationship), self-concept, intense physical activity (PA) outside school. The results indicate that academic performance correlates positively with the concentration variable (Z = 0.177, *p* = 0.012), with the NPB dimension of Relationship (Z = 0.259, *p* < 0.001), with Academic Self-Concept (Z = 0.308, *p* < 0.001) and with the practice of moderate or intense PA (Z = 0.262, *p* < 0.001).

### 3.5. Variables That Influence the Development of AP in Spelling

Table 5 identifies studied variables that may influence the development of AP in spelling in young people who have carried out the CF-AB intervention program. In the multiple linear regression model, the results show that the variables academic self-concept, relationship, and practicing intense PA outside school significantly predict academic performance development in spelling: F (4.198) = 12.280. Taking all these variables, 20% of the variance of AP in spelling is explained, excluding the concentration variable from this profile.

The multiple linear regression model found that the young people, who participated in the CF-AB intervention program, who obtained better AP in spelling are those who practice more intense physical activity outside school, those who have a good academic self-concept, and those who relate better with their peers.

## 4. Discussion

Considering that the child population spends much of its time in schools performing mostly sedentary tasks focused on the acquisition of knowledge, skills, and abilities [9], the CF-AB strategy facilitates a reduction in sedentary levels in the student population and an improvement in cognitive functions related to learning processes [6,7] along with an improvement in the neurocognitive and psychoemotional development of young people [8]. However, the existing literature on the incidence of CF-AB in AP is uncertain, which may be due, as Nieto Martín [11] suggests, to the fact that AP is multifactorial, composed of personal variables (cognitive variables, study habits, motivation, attention, concentration, self-concept, emotions) and social variables (social environment, socioeconomic context, and socio-democratic dimensions).

In view of the above background, the objectives of this study were (1) to describe and analyze the effects of the implementation of a program of curricular active breaks (CF-AB), associated with spelling learning, on the personal variables of attention and concentration and (2) to analyze the relationship between intense physical activity (PA) and the personal variables of attention, concentration, academic self-concept and the need to relate to others, variable that is included in the social variable, on spelling performance after this intervention.

To respond to the first purpose of the research, the results of the research show that, after intervention with CF-AB, the values of the attention and concentration variables increased more in the experimental group than in the control group, as well as the studies of Ma et al. [39] and Magistro et al. [22], although the differences were not statistically significant. These results coincide with the research of Arribas-Galarraga et al. [17], in which the incidence of CF-ABs in secondary school students (mean age = 12.13 years) is analyzed. The results after the intervention showed significant differences (*p* < 0.01) and a greater increase in the attention variable in the intervention group than in the control group, although differences between groups were not significant. In a similar way, the results of the present study indicate, as in the study made by Bartholomew et al. [19], that students’ attention is higher after active breaks compared to attention levels when they are engaged in sedentary activities.

Regarding concentration, both groups showed a slight improvement, but without statistical significance. However, it should be noted that the results of the current study agree with studies by Contreras Jordán et al. [40], Peiris et al. [43], Mercader [45], and Scholz et al. [44], where a trend in the improvement of concentration levels is observed after the implementation of programs based on active breaks. On the other hand, curiously, coinciding with Mercader [45], it is worth noting that concentration does not seem to explain improvement in AP, since, according to this author, concentration is not a predictor of academic performance.

Considering the slight difference in favor of attention and concentration variables in the experimental group, after the 8 week intervention, we can appreciate the need to implement intervention for longer periods of time, as these results may reflect an incipient improvement that could increase with the extension in time. When implementing active breaks in the classroom, it is important to consider the review by Pastor-Vicedo et al. [42], who point out that the most favorable characteristics are duration and intensity in obtaining better results in the variables of attention, concentration, and AP. They conclude that interventions should be implemented with duration of 5–10 min and that PA should be performed at a vigorous intensity. However, the positive trend of the experimental group in the improvement of attention and concentration suggests that the application of active breaks may have a positive impact on this group; therefore, we insist on further study to obtain more conclusive results.

When analyzing the incidence of CF-AB on the AP variable, the results showed a decrease in the AP score in both groups after the intervention. As mentioned above, there is controversy among the reviewed studies. Although some studies such as Contreras Jordán et al. [40] and Peiris et al. [43] support the improvement of AP after intervention with AB, the results of the current study coincide with other studies in which the desired AP was also not obtained [17,25,26,27,28,29,30,31,32,33,34]. Taking Nieto Martín’s [11] case into account, the results obtained in this paper may be because AP is a multifactorial variable influenced by personal and social factors. In future research, special attention should be paid, among others, to possible stressors of AP (e.g., school evaluation period).

Considering the multifactorial nature of AP, the present research analyzes the relationship between intense physical activity and the personal variables of attention, concentration, academic self-concept, and the social variable of the social environment through the basic psychological needs variable that encompasses the dimensions of autonomy, relationship satisfaction, and competence satisfaction. In line with other research, this study also found that AP positively correlates with the “relatedness” dimension of basic psychological needs [56,57,59], academic self-concept [48,49,50,51], and moderate-to-vigorous physical activity outside of school. These findings suggest that maintaining relationships with others, having a high academic self-concept, and engaging in vigorous physical activity may influence students’ academic performance. In order to know the profile of the student who obtains better results in spelling performance after an intervention with CF-AB, a regression analysis was performed. The results suggest that students who obtained a better academic performance in spelling practiced more intense PA outside school, had a good academic self-concept, and maintained more satisfactory social relationships with their peers.

Future research studies may pay special attention to the number of sessions per week, the prolongation of the same during the school year, the intensity and duration of the activity, and the intervention of active breaks. In addition, it is also suggested to pay attention to possible AP stressors related to the multifactoriality of AP. Thus, to analyze the incidence of active breaks on AP, it is necessary to consider the personal variables (attention, concentration, self-concept, and motivation) and social variables (social environment, socioeconomic context, and the socio-democratic dimension) involved in AR.

Academic performance (AP) in Spelling was the curricular content analyzed in this study, presenting unfavorable results after the intervention. The results lead us to reflect on the weaknesses of the study. To this effect, it should be considered that although the scientific literature is solid regarding the relationship between the multiple benefits of PA practice on physical and psychological health and the multiple benefits on the neurocognitive and psychoemotional development of students [8], the existing results on the incidence of active breaks on attention, concentration, and AP should be interpreted with caution due to the variety of interventions in terms of duration, type, and intensity [42]. Therefore, the results on the incidence of active breaks on attention, concentration and AP variables in the present study should be interpreted with caution. It is necessary to investigate further in future research on the multifactorial nature of AP, since it is an important aspect to take into account, especially the stressors of AP in addition to the PA variable.

## 5. Conclusions

This study’s results show a slight trend toward improved attention and concentration after an 8-week implementation of a CF-AB program. This positive trend suggests the need for a longer implementation over time, with interventions of 5–10 min and vigorous intensity to obtain the most effective results.

After the intervention, the unfavorable results of academic performance (AP) in Spelling lead us to reflect on the multifactorial nature, especially its stressors of academic performance the duration, type, and intensity of active breaks.

On the other hand, considering the relationship between the analyzed variables, the conclusion is that relational support, academic self-concept, and intense physical activity can influence students’ academic performance. The results show that the more intense the physical activity, the better the academic self-concept and the greater the relational support, the higher the students’ academic performance. However, it is worth noting that concentration does not seem to explain the improvement in academic performance.

We have obtained the profile of those who have most improved their AP in spelling among the participants in the CF-AB program in this study. These are students who engage in intense physical sports practice outside school, who show a high academic self-concept, and who best perceive that they relate to others.

Finally, these findings highlight the importance of further research on the effects of CF-AB by addressing socioemotional and physical activity factors on the improvement of academic performance, which may have significant implications for the design of educational interventions and student support programs. We insist on the need to explore other variables and factors that may also influence academic performance and contribute to a more complete understanding of this multifactorial phenomenon in future research.

## Figures and Tables

**Table 1:** D2 test scores (attention; concentration).

Group		Attention		Concentration	
		Pre	Post	Pre	Post
Control	M	352.04	388.22	128.80	144.42
(*n* = 114)	DT	75.490	82.939	38.431	45.961
	Max	559	591	206	260
	Min	12	101	2	4
	z	−4.668		−4.212	
	*p*	<0.001 *		<0.001 *	
Experimental	M	358.54	396.62	134.64	149.84
(*n* = 199)	DT	67.206	70.280	29.748	37.578
	Max	535	612	213	266
	Min	104	185	29	9
	z	−9.151		−7.128	
	*p*	<0.001 *		<0.001 *	

* *p* < 0.001.

**Table 2:** Scores obtained in academic achievement tests.

Group	RA		
		Pre	Post
Control	M	121.26	107.47
(*n* = 114)	DT	31.668	32.968
	Max	220	196
	Min	67	25
Experimental	M	121.13	118.01
(*n* = 199)	DT	38.797	41.646
	Max	257	333
	Min	1	0

**Table 3:** Self-concept (academic), basic psychological needs (relationship), and amount of physical activity.

Group		Self-Concept: Academic	NPB: Ratio	Moderate or Severe PA (Hours/Week)
Control	M	6.804	17.02	3.80
(*n* = 114)	DT	1.673	8.640	1.631
	Max	9.90	31	7
	Min	0.15	0	1
Experimental	M	6.847	23.57	3.80
(*n* = 199)	DT	2.03	5.905	1.551
	Max	9.90	33	7
	Min	0	5	1

**Table 4:** Academic performance correlation (post)—intense physical activity outside school, concentration (post), attention (post), relationship, self-concept.

Variable		(1)	(2)	(3)	(4)	(5)	(6)
(1) Academic Performance Post-test	Correlation	1					
	P						
(2) Attention Post-test	Correlation	0.126	1				
	P	0.077					
(3) Post-test Conception	Correlation	0.177 *	0.833 **	1			
	P	0.012	<0.001				
(4) Basic Psychological Necessities: Relationship	Correlation	0.259 **	−0.118	−0.001	1		
	P	<0.001	0.097	0.988			
(5) Self-concept: Academic	Correlation	0.308 **	0.192 **	0.280 **	0.181 *	1	
	P	<0.001	0.007	<0.001	0.011		
(6) Moderate or severe PA (Hours/week)	Correlation	0.262 **	−0.015	0.077	0.074	0.031	1
	P	<0.001	0.836	0.282	0.297	0.660	

* Correlation is significant at the 0.05 level (bilateral). ** Correlation is significant at the 0.01 level (bilateral).

**Table 5:** Regression.

		B	ER **	β	t	*p*-Value	F/R^2^
Step 1	Self-concept Academic	6.313	1.390	0.308	4.540	1	F (1.198) = 20.612 R^2^ = 0.095
Step 2	Self-concept Academic	5.747	1.445	0.280	3.978	0.001	F (2.198) = 11.338 R^2^ = 0.104
	Concentration	0.109	0.078	0.099	1.401	0.163	
Step 3	Self-concept Academic	4.877	1.438	0.238	3.392	0.001	F (3.198) = 11.341 R^2^ = 0.149
	Concentration	0.123	0.078	0.111	1.607	110	
	Relation	1.521	0.475	0.216	3.205	0.002	
Step 4	Self-concept Academic	4.896	1.395	0.239	3.504	0.001	F (4.198) = 12.280 R^2^ = 0.202
	Concentration	0.103	0.074	0.093	1.381	0.169	
	Relation	1.398	0.462	0.198	3.027	0.003	
	Actv.phys.out	28.716	7.963	0.233	3.606	0.001	

** ER: Standard error.

## Data Availability

The data presented in this study are available on request from the corresponding author.
